# The Role of Electrostatic Interactions in Binding of Histone H3K4me2/3 to the Sgf29 Tandem Tudor Domain

**DOI:** 10.1371/journal.pone.0139205

**Published:** 2015-09-30

**Authors:** Bas J. G. E. Pieters, Erik Meulenbroeks, Roman Belle, Jasmin Mecinović

**Affiliations:** Institute of Molecules and Materials, Radboud University Nijmegen, Nijmegen, The Netherlands; Universität Stuttgart, GERMANY

## Abstract

Several reader domain proteins that specifically recognize methyllysine-containing histones contain the negatively-charged aspartate or glutamate residues as part of the aromatic cage. Herein, we report thermodynamic analyses for the recognition of histone H3K4me3 and H3K4me2 by the tandem tudor domain of Sgf29 and its recognition site variants. Small uncharged and large aromatic substitutions on the Asp266 site resulted in a significant decrease in binding affinities for both H3K4me3 and H3K4me2, demonstrating the role of the negative charge of Asp266 in the readout process by Sgf29. This study emphasizes the essential contribution of electrostatic interactions to the overall binding affinity, and reveals that the underlying mechanisms for the recognition of Kme2/3 depend on the composition and arrangement of the aromatic cage.

## Introduction

In order to structure the vast amount of genetic material stored within the cell, several mechanisms of DNA condensation have evolved in eukaryotic organisms. The first level of condensation is achieved by wrapping DNA around a protein assembly of eight histone proteins to form the nucleosome[1]. The histone tails protruding from this assembly are especially accessible for various posttranslational modifications (PTM) such as acetylation, phosphorylation and methylation[2]. These PTMs have been found to be involved in processes such as gene activation, gene transcription and chromatin condensation. Not surprisingly, due to this close relation of epigenetics to cellular function, alterations in function of proteins involved in epigenetic gene regulation have been associated with human disease, most notably various cancers[3,4].

One of the most widespread histone PTMs is lysine methylation, which can be found in three different methylation states: monomethylated (Kme1), dimethylated (Kme2) and trimethylated (Kme3). The process of lysine methylation is controlled by three types of proteins, namely histone lysine methyltransferases (writers), histone lysine demethylases (erasers), and histone lysine reader domain proteins (also known as chromatin effectors), that are capable of specifically recognizing posttranslationally-modified histones[5,6]. A common feature of readers that specifically recognize Kme3 and Kme2 is that they possess a methyllysine recognition site referred to as the aromatic cage. Such an aromatic cage typically consists of 2–4 aromatic amino acid residues, which are capable of interacting with the positively-charged Kme2 and Kme3 side-chain, presumably via a combination of cation-π, hydrophobic and electrostatic interactions[7]. Aromatic cages flanking the Kme2/3 on two or three sides are referred to as half cages, whereas those flanking the Kme2/3 at four sides are referred to as full cages.[7,8]. Half cages usually contain an additional negatively-charged amino acid (aspartic acid or glutamic acid) instead of an aromatic amino acid, as is the case with full cage binding sites. Comparative analyses determined that, in general, binding affinities follow the trend of Kme3 > Kme2 > Kme1 > K[7]. Substitution studies on residues that constitute the readers’ aromatic cages demonstrated the involvement of individual amino acids in associations with Kme2 or Kme3[7,8].

**Fig 1:**
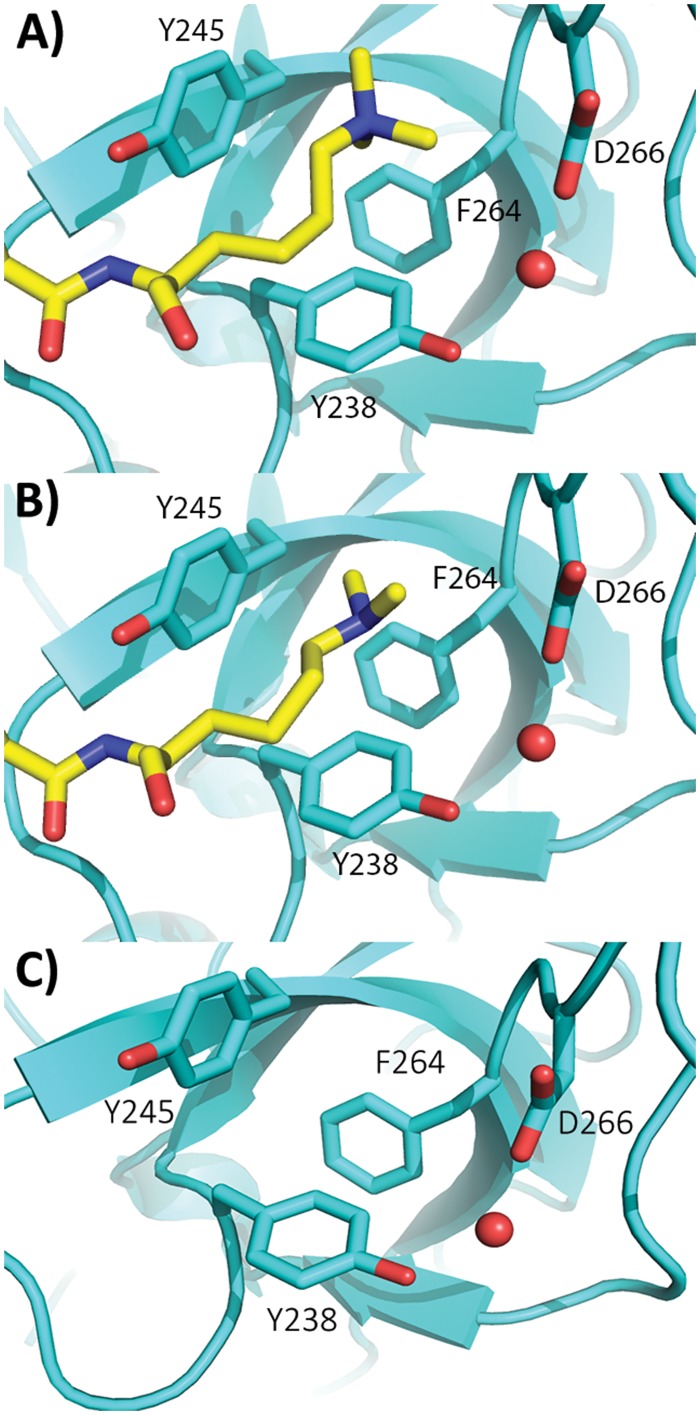
Crystal structure of Sgf29-H3K4me3 (A, PDB ID: 3ME9), Sgf29-H3K4me2 (B, PDB ID: 3MET) and the apo form of Sgf29 (C, PDB ID: 3MEW). Sgf29 and H3K4me2/3 histone peptide are shown in cyan and yellow, respectively.

Sgf29 (SAGA-associated Factor 29) is a subunit of the SAGA (Spt-Ada-Gcn5 acetyltransferase) complex, a chromatin modifying complex, which is capable of acetylating and deubiquitinating histone proteins.[9,10]. Crystallographic work has shown that Sgf29 contains a tandem tudor domain with each tudor domain having a negatively-charged pocket capable of binding H3A1 and an aromatic cage for the recognition of H3K4me2/3 residues (Fig 1).[11]. The aromatic cage is composed of 3 aromatic amino acid residues and a negatively-charged aspartate (Y238, Y245, F264, and D266), with F264 being positioned at the edge of the recognition site. Thermodynamic analyses have confirmed the requirement of the H3A1 recognition site for adequate binding of H3K4me3 peptides to Sgf29 and that only the first four N-terminal amino acid residues (ARTKme3) are essentially required for binding of H3K4me3 peptides to the Sgf29 reader domain[12].

This study examines the molecular basis for the involvement of the negatively-charged aspartate/glutamate in the biomolecular recognition of Kme2/3-containing histone peptides using Sgf29 as a reader domain protein. We chose the tandem tudor domain of Sgf29 for this study for the following reasons: 1) the Kme2/3 recognition site contains three aromatic residues and one negatively-charged Asp266; 2) X-ray crystal or NMR solution structures for Sgf29, Sgf29-H3K4me2 and Sgf29-H3K4me3 complexes have been solved (Fig 1); 3) binding affinities in low micromolar range can be determined by isothermal titration calorimetry (ITC); 4) wild-type Sgf29 and its variants can be obtained in quantities necessary for further biochemical and biophysical studies. In order to probe the role of Asp266 in the Sgf29-H3K4me2/3 interactions, the D266 residue was substituted by various amino acids using site directed mutagenesis. Firstly, the role of D266 in the Sgf29-H3K4me2/3 binding event was investigated by D266 substitution, using amino acid residues that vary in length and charge. Secondly, D266 was substituted by various aromatic residues in order to study the effect of the potential full cage-like structure on the association between Sgf29 and H3K4me2/3. Here we report the systematic thermodynamic analyses for the association of H3K4me2/3 histone peptides to Sgf29 and its aromatic cage variants for the first time, with a special focus on elucidating the role of Asp266 in the Sgf29-H3K4me2/3 interactions.

## Results & Discussion

**Fig 2:**
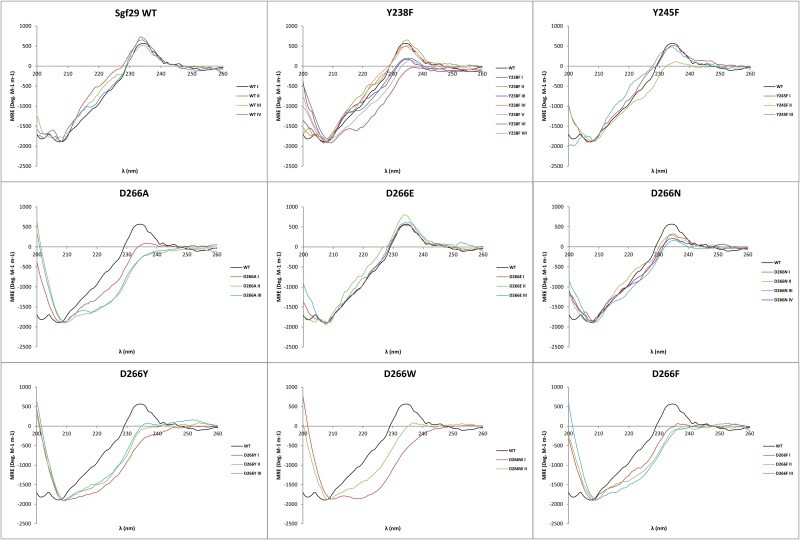
CD spectra of various expressions of wild-type Sgf29 its D266 variants, and the Y238F and Y245F variants. CD experiments were carried out at the concentration of 0.1 mg ml^-1^ in 10 mM sodium phosphate buffer (pH 7.5).

Fmoc-Kme2-OH and Fmoc-Kme3-OH were incorporated in 10-mer histone 3 peptides (peptide sequences: ARTKme2QTARKS and ARTKme3QTARKS) on position 4 using solid-phase peptide synthesis. The two peptides were purified by R_f_-C_18_ preparative HPLC and the lyophilized peptides were used for binding studies (S1 and S2 Figs). Wild-type Sgf29 and its recognition site variants were expressed in *E*. *coli* and subsequently purified (S3 Fig). In order to determine whether the variations introduced at the D266 site of Sgf29 caused structural perturbations, CD experiments were initially carried out on the recombinantly expressed wild-type Sgf29 (hereafter referred to as WT Sgf29) and its variants (Fig 2). Multiple batches of WT Sgf29 and each of its variants were produced in order to take into the account the batch-to-batch variation of proteins (Fig 2). Collectively, the CD data showed that WT and D266E Sgf29 have very similar CD spectra, whereas D266N has a slightly altered spectrum. D266A, D266F, D266Y and D266W variants displayed significantly different spectra when compared to WT Sgf29. These results are possibly due to the fact that D266 is positioned in an unstructured loop region of the reader domain and has a potential to interact with the adjacent Y238 *via* a water-mediated hydrogen bond, as suggested by structural analyses of the apo and holo forms of Sgf29 (Fig 1). Upon abrogation of this hydrogen bond, the unstructured D266 loop region might adopt alternative conformations resulting in altered tertiary protein structure. In order to test this hypothesis, we mutated the Y238 residue, which is also located in an unstructured region of Sgf29, into phenylalanine, which does not have the ability to directly participate in hydrogen bonding. From the seven batches of Y238F proteins we prepared, a broad range of CD spectra have been observed, ranging from wild-type like to a distinctly altered spectrum.

**Fig 3:**
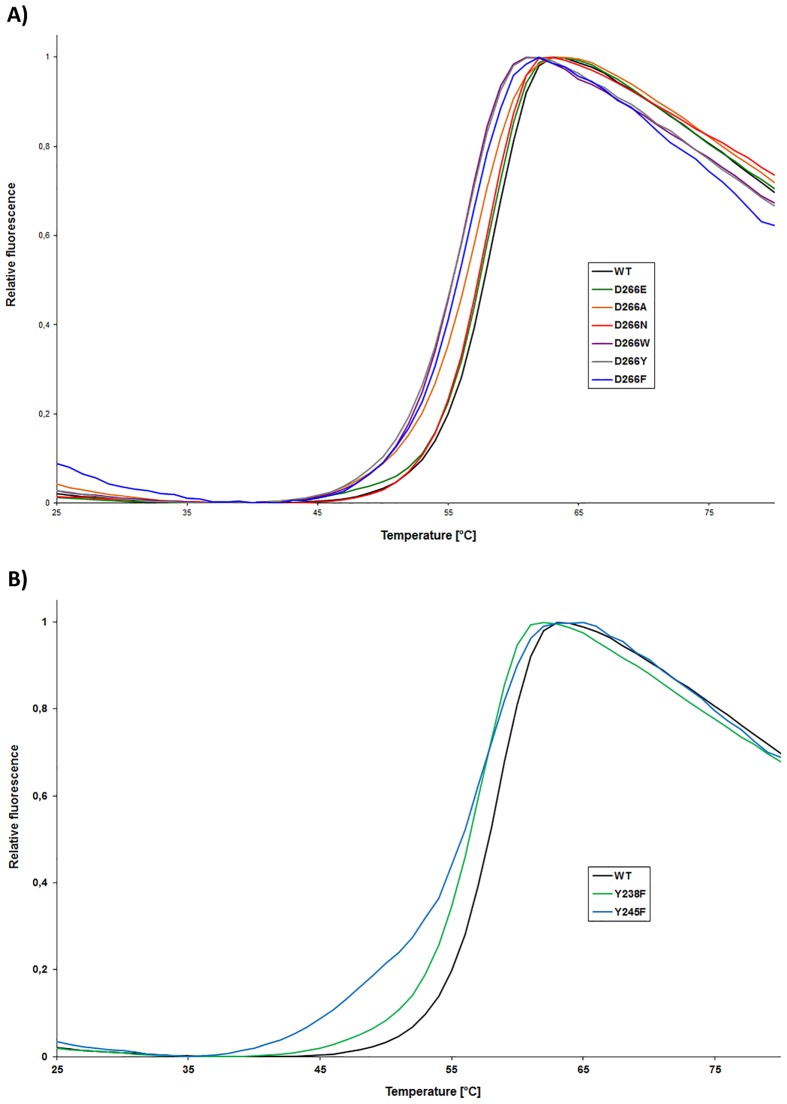
Tm curves of A) wild-type Sgf29 and its D266 variants and B) Sgf29 and its Y238 and Y245 variants.^[a]^

Additionally, differential scanning fluorimetry (DSF) was employed as a secondary method to determine the structural stability of the Sgf29 variants (Fig 3, Table 1). Using DSF, WT Sgf29 was shown to have a Tm of 57.8°C. Variant proteins have similar or decreased Tm values when compared to WT Sgf29, ranging from 53.9–57.7°C. The introduction of the negatively-charged Glu at the D266 site gave indistinguishable Tm values relative to WT Sgf29. In addition, the introduction of aromatic residues only had a small influence on Tm, as substitution of D266 with Trp, Phe or Tyr only decreased Tm by 2.3, 3.3 or 3.9°C, respectively. Collectively, all Tm values can be considered to be within close proximity to the Tm for WT Sgf29. These data, combined with CD spectra indicate that D266A, F, Y and W variants have altered tertiary structures, which do not substantially affect the stability of the variant proteins. Because the D266 residue is located in an unstructured region on the surface of the reader domain, alterations made to this residue do not appear to affect the more structurally and thermodynamically stable β-sheet rich regions. These structured regions make up the major part of the Sgf29 reader domain, explaining the subtle changes in Tm values for the D266X variants. Similarly, we observed similar Tm values for all Y238F variants that display altered and unchanged CD spectra relative to WT Sgf29, suggesting that different conformations of the D266 containing loop region do not substantially affect the stability of this variant. Y245F variant displays similar Tm value as the related Y238F.

**Table 1:** Tm values for Sgf29 and its variants as determined by DSF.^[a]^

Sgf29	Tm (°C)
**WT**	57.8 ± 0.1
**D266E**	57.7 ± 0.1
**D266A**	55.8 ± 0.3
**D266N**	56.4 ± 0.1
**D266W**	55.5 ± 0.1
**D266Y**	54.5 ± 0.2
**D266F**	53.9 ± 0.5
**Y238F**	56.5 ± 0.2
**Y245F**	56.4 ± 0.1

^[a]^ Tm as measured by DSF ± SD (Measured in triplicate)

**Fig 4:**
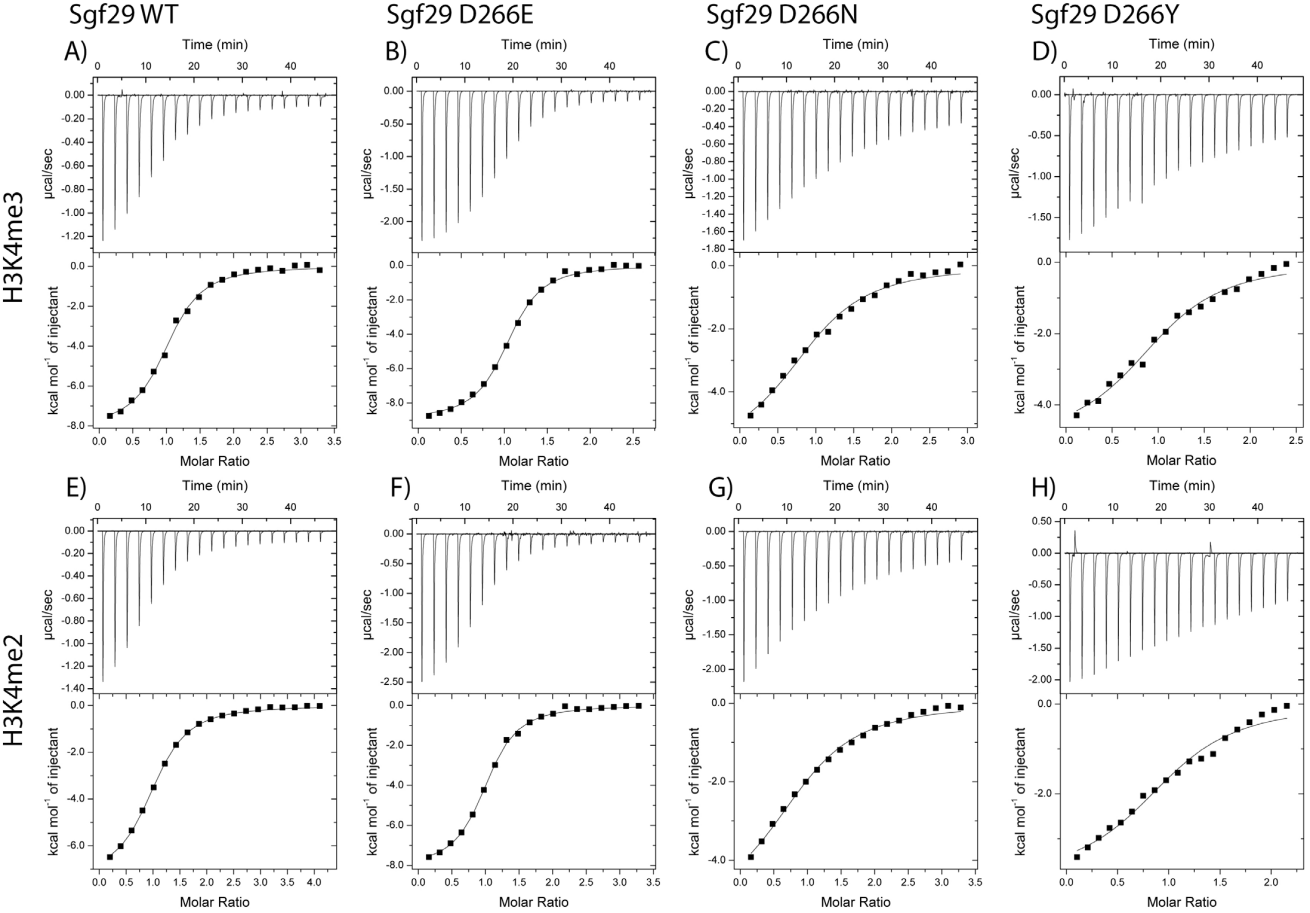
Thermodynamic analyses of Sgf29-H3K4me2 and Sgf29-H3K4me3 interactions. Representative ITC experiments showing the titration of H3K4me3 (top row, A-D) and H3K4me2 (bottom row, E-H) peptides to Sgf29 (first column, A,E), D266E (second column B, F), D266N (third column, C, G) and D266Y (fourth column, D, F).

Recognition of 10-mer H3K4me3 and H3K4me2 histone peptides by WT Sgf29 and its D266 variants was examined by isothermal titration calorimetry (ITC). ITC provided full thermodynamic descriptions (i.e. dissociation constant *K*_d_, free energy of binding Δ*G*°, enthalpy of binding Δ*H*°, and entropy of binding -TΔ*S*°) for the association of reader-histone complexes (Table 2, Fig 4). Consistent with previous studies, thermodynamics of interactions between H3K4me3 and Sgf29 showed that the association is enthalpy-driven (Δ*H*° = -8.1 kcal mol^-1^), whereas the entropy of binding is slightly unfavorable (-TΔ*S*° = 0.6 kcal mol^-1^)[12]. H3K4me2 histone peptide exhibits slightly lower binding affinity than H3K4me3 for binding to WT Sgf29 (*K*_d_ = 4.7 μM for H3K4me2 vs. 3.1 μM for H3K4me3). Thermodynamic analyses furthermore demonstrated that there is an enthalpy-entropy compensation for binding of H3K4me3 and H3K4me2 to WT Sgf29. Δ*H*° is more favorable for the formation of Sgf29-H3K4me3 complex relative to Sgf29-H3K4me2 by 0.9 kcal mol^-1^, while -TΔ*S*° is less favorable by 0.7 kcal mol^-1^. Similar observations have been reported for other reader domain proteins, including BPTF and JARID1A, which specifically recognize H3K4me3 and H3K4me2 [13,14].

**Table 2:** Thermodynamic parameters for binding of histone H3K4me2 and H3K4me3 peptides to Sgf29 variant proteins.^[a]^

Sgf29	*K*_d_ (μM)	Δ*G*° (kcal mol^-1^)	Δ*H*° (kcal mol^-1^)	-TΔ*S*° (kcal mol^-1^)	*K*_d_ (μM)	Δ*G*° (kcal mol^-1^)	Δ*H*° (kcal mol^-1^)	-TΔ*S*° (kcal mol^-1^)
	**H3K4me2**				**H3K4me3**			
**WT**	4.7 ± 0.3	-7.3 ± 0.1	-7.2 ± 0.2	-0.1 ± 0.2	3.1 ± 0.3	-7.5 ± 0.1	-8.1 ± 0.2	0.6 ± 0.2
**D266E**	5.6 ± 1.5	-7.2 ± 0.2	-7.9 ± 0.4	0.7 ± 0.6	3.9 ± 0.3	-7.4 ± 0.1	-9.0 ± 0.1	1.6 ± 0.1
**D266A**	38 ± 4	-6.0 ± 0.1	-6.1 ± 0.2	0.1 ± 0.1	25 ± 3	-6.3 ± 0.1	-6.4 ± 0.1	0.1 ± 0.1
**D266N**	44 ± 5	-6.0 ± 0.1	-5.4 ± 0.2	-0.6 ± 0.2	25 ± 2	-6.3 ± 0.1	-6.2 ± 0.1	-0.1 ± 0.1
**D266W**	NB^[b]^	NB	NB	NB	NB	NB	NB	NB
**D266Y**	38 ± 4	-6.0 ± 0.1	-3.9 ± 0.2	-2.1 ± 0.2	28 ± 4	-6.2 ± 0.1	-4.8 ± 0.2	-1.4 ± 0.3
**D266F**	57 ± 3	-5.8 ± 0.1	-2.6 ± 0.2	-3.2 ± 0.1	44 ± 5	-5.9 ± 0.1	-3.4 ± 0.2	-2.5 ± 0.1
**Y238F**	6.8 ± 0.4	-7.1 ± 0.1	-7.0 ± 0.1	-0.1 ± 0.2	3.5 ± 0.2	-7.4 ± 0.1	-8.1 ± 0.1	0.7 ± 0.1
**Y245F**	19 ± 2	-6.4 ± 0.1	-4.5 ± 0.1	-1.9 ± 0.1	14 ± 1	-6.6 ± 0.1	-5.9 ± 0.1	-0.7 ± 0.1

^[a]^ As measured by ITC ± SD (2–4 repeats).

^[b]^ NB = No Binding (*K*_d_ > 200 μM).

We then investigated the importance of the negatively-charged D266 on the recognition of H3K4me2/3 histones. D266 is located in the Sgf29’s Kme2/3 recognition site, complementing the aromatic cage that consists of Y238, Y245 and F264 (Fig 1). Substitution of D266 by glutamic acid (D266E), an amino acid of one-carbon longer side-chain and the same negative charge as aspartic acid, showed virtually indistinguishable binding affinities for H3K4me3 and H3K4me2, respectively. Interestingly, D266E substitution results in more favorable Δ*H*° (by -0.9 kcal mol^-1^), which is fully compensated by more unfavorable -TΔ*S*° (by 1.0 kcal mol^-1^) for both H3K4me3 and H3K4me2. This result can be rationalized by increased flexibility of glutamate relative to aspartate in the unbound state, which becomes substantially restricted upon the formation of a complex with H3K4me2/3.

Substitution of the negatively-charged aspartate D266 by neutral and smaller alanine (D266A) resulted in a significant reduction in binding affinities for H3K4me3 (8-fold) and H3K4me2 (7-fold), suggesting the essential contribution of the negative charge of D266 to the overall binding affinities. The observed similar decreases in Δ*G*° values are a result of more unfavorable Δ*H*° for H3K4me3 (1.7 kcal mol^-1^) and H3K4me2 (1.1 kcal mol^-1^), implying that the underlying mechanism for the loss of binding affinity for D266A is similar for both peptides.

When D266 was substituted by the uncharged asparagine (D266N), it was observed that there was a decrease, similar to the D266A substitution, in binding affinity for H3K4me3 (8-fold) and H3K4me2 (9-fold) when compared to the WT Sgf29. The decrease in Δ*H*° for H3K4me3 was observed to be 2.9 kcal mol^-1^, which was compensated by an increase in entropy of 0.7 kcal mol^-1^ resulting in a Δ*G*° of -6.3 kcal mol^-1^. For H3K4me2 the decrease in Δ*H*° was observed to be 1.8 kcal mol^-1^ and compensated by an increase in-TΔ*S*° of 0.5 kcal mol^-1^ resulting in a Δ*G*° of -6.0 kcal mol^-1^. When compared to the WT Sgf29, D266A and D266E variants, these data indicate that the overall negative charge on D266 is responsible for the observed favorable binding affinities for H3K4me2/3, the length of the side-chain being less important.

Having shown that D266 has an important role in the strong recognition of H3K4me3 and H3K4me2 histone peptides, we aimed to provide further explanation for such observations. Based on crystallographic analyses of Sgf29-H3K4me3 and Sgf29-H3K4me2 complexes, D266 could contribute into the overall binding affinity in three possible ways: i) favorable direct electrostatic interactions with Kme2/3, ii) favorable H-bond network that involves a water molecule and Y238, and iii) favorable salt-bridge with R8 of histone 3.

Firstly, our thermodynamic analyses show that all D266X variations affect the binding affinities for H3K4me3 and H3K4me2 in very similar fashion (i.e. ΔΔ*G*°, ΔΔ*H*°, and-TΔΔ*S*° values for association of Kme3 to D266X relative to WT Sgf29 are similar to ΔΔ*G*°, ΔΔ*H*°, and -TΔΔ*S*° values for Kme2-binding). These results imply that D266 likely contributes to binding of Kme3 and Kme2 in the same way. The distance between the negatively-charged oxygen of D266 and the hydrogen atoms of the positively-charged CH_3_N^+^ of Kme3 and Kme2 is about 3.0 Å, which is within the van der Waals contact distance. Notably, the hydrogen atom bonded to nitrogen in ^+^NHMe_2_ is positioned away from the D266 side-chain, suggesting the absence of direct H-bonding or salt-bridge between ^+^NHMe_2_ and D266 (there are two conformations of Sgf29-H3K4me2 structure, both having NH^+^ positioned away from the D266 site; PDB ID: 3MET). Thus, in the case of Sgf29, it is likely that interactions between D266 and the Kme2/3 side-chain are predominantly electrostatic in nature.

**Fig 5:**
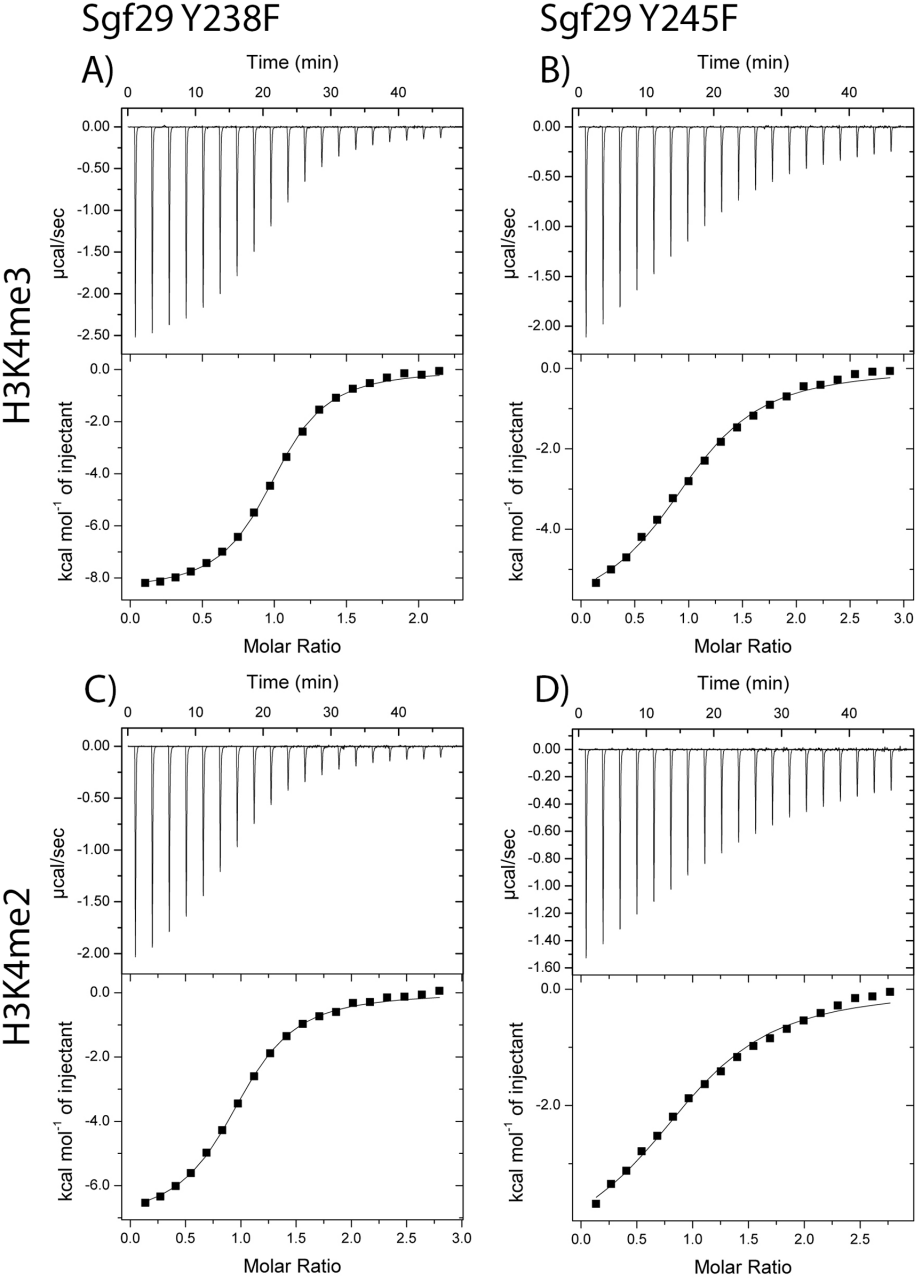
Representative ITC experiments displaying binding of H3K4me3 and H3K4me2 to Y238F and Y245F Sgf29 variants. A) H3K4me3-Y238F, B) H3K4me3-Y245F, C) H3K4me2-Y238F and D) H3K4me2-Y245F.

The second possibility for the observed involvement of D266 in recognition of H3K4me2/3 is that the network of H-bonding between Y238-water-D266 is abrogated in D266A, which consequently results in an overall decrease in Δ*G*°. In order to test this hypothesis, a simple variation was introduced at the Y238 position. By substituting tyrosine by phenylalanine (Y238F) the ability of Y238 to form a hydrogen bond is abrogated. Importantly, ITC data on Y238F variants that displayed both altered or similar CD spectra relative to WT Sgf29, showed indistinguishable thermodynamic profiles for H3K4me3 and H3K4me2 binding, when compared to WT Sgf29 (Fig 5). These results suggest that even if the Sgf29 variants display altered conformations in their apo form, they might have the ability to adopt a properly folded aromatic cage WT-like structure in the complexed Sgf29-H3K4me2/3 form. In contrast to Y238F, Y245 substitution (Y245F) resulted in a reduction in binding affinities. H3K4me2/3 binding became entropically more favorable, but this was offset by a decrease in enthalpy of binding resulting in a 4.5- and 4.0-fold reduced binding affinities for H3K4me3 and H3K4me2, respectively. This result can be explained by the fact that Y245 forms a H-bond with the Kme2/3’s backbone NH of the amide group (Fig 1). When this hydrogen bonding potential is abolished (as in Y245F), the enthalpy of binding is reduced, thus resulting in less favorable binding affinity. In contrast, Y238 interacts with D266 *via* a water-mediated hydrogen bond. This interaction likely helps to stabilize the unstructured D266 loop, whereas the tyrosine’s hydroxyl group is not directly involved in H3K4me3 and H3K4me2 peptide binding. Overall, these data, supported by structural analyses of the free and complexed forms of Sgf29, illustrate that the water-mediated H-bonding between D266 and Y238 helps to stabilize Sgf29 and enables the formation of the static aromatic cage for the recognition of H3K4me2/3. The lack of D266-water-Y238 hydrogen bonding network, however, is not determining the binding affinity between Sgf29 and H3K4me2/3, because Y238F variants that display altered CD spectra exhibit similar thermodynamics of interactions to the unaltered counterparts. These data suggest that protein variants with alternative loop folds have the ability to form a properly folded aromatic cage that resembles the WT aromatic cage in the bound Sgf29-H3K4me2/3 complexes. Recent work by Ali *et al*. supports this view, as the crystallographic analyses of the apo and holo forms of the PHD domain of MLL5 showed that the unbound and bound forms of the protein exhibit different conformations of the N-terminal loop region[15]. The binding of H3K4me3 substrate to MLL5 is associated with a large conformational change of the unstructured loop that contains the negatively-charged D128, which is part of the aromatic cage/recognition site and directly interacts with the Kme3 side chain. Thus, the ability of the free protein to undergo the conformational change is a prerequisite for the energetically favorable association with its H3K4me3 substrate. In regard to our results on Sgf29, it is possible that the D266X variants, although most of them bear different conformations of the loop region in the free state, may have an ability to adopt the conformation in the bound state that is similar to the conformation of the WT Sgf29.

In line with these results, more detailed analyses of the crystal or solution structures of the apo and holo forms of epigenetic reader domain proteins that contain the aromatic cage for specific binding of methylated lysines show that in many cases aromatic residues and negatively-charged Asp/Glu residues are positioned in the unstructured loop regions (although these residues are also found in α-helices and β-sheets)[7]. It is noteworthy that substitutions of these residues typically result in decreased binding affinities for associations with Kme2/3-containing histones. The observed weaker binding has often been attributed to the lack of favorable non-covalent interactions, although it is important to consider that structural changes may also influence the binding affinities.

**Fig 6:**
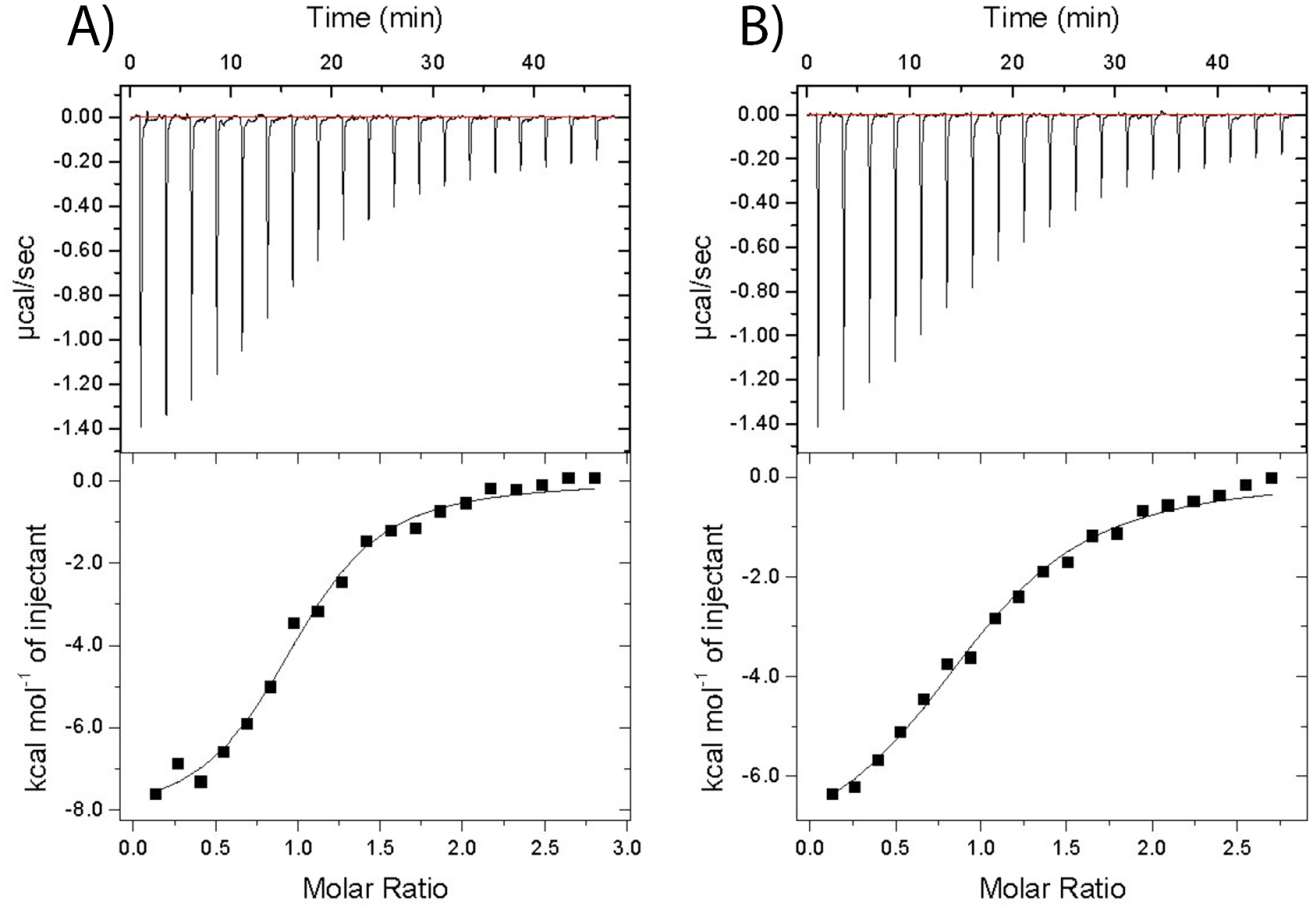
ITC experiments showing binding of A) ARTKme3QTAGKS and B) ARTKme3QTA to WT Sgf29. Thermodynamics of binding for A) *K*_d_ = 4.0 ± 0.6 μM, Δ*G*° = - 7.4 ± 0.1 kcal mol^-1^, Δ*H*° = - 8.0 ± 0.1 kcal mol^-1^,-TΔ*S*° = 0.6 ± 0.1 kcal mol^-1^ and for B) *K*_d_ = 9.0 ± 0.5 μM, Δ*G*° = - 6.9 ± 0.1 kcal mol^-1^, Δ*H*° = - 7.8 ± 0.1 kcal mol^-1^,-TΔ*S*° = 0.9 ± 0.1 kcal mol^-1^.

The involvement of the potential salt-bridge between the negatively-charged D266 and positively-charged R8 of 10-mer H3K4me3 and H3K4me2 peptides was also examined (PDB ID: 3ME9, 3MET). 10-mer H3K4me3 peptide that contains G instead of R at position 8 bound to WT Sgf29 with a *K*_d_ of 4 μM, similar to the native 10-mer peptide (Fig 6A). In addition, 7-mer peptide ARTKme3QTA that lacks the last three residues, including the eighth residue R8, bound to WT Sgf29 with *K*_d_ value of 9 μM, demonstrating the 3-fold decrease of the binding affinity relative to 10-mer H3K4me3 peptide (Fig 6B). These data suggest that it is unlikely that potential D266-R8 salt-bridge contributes to a significant extent to the overall favorable association between H3K4m2/3 and Sgf29.

Next, with the aim to construct a full cage-like reader module, D266 was substituted by the aromatic amino acids tryptophan, tyrosine or phenylalanine (D266W, D266Y, or D266F, respectively). When a D266W substitution was introduced, however, binding of H3K4me3 and H3K4me2 was abrogated. These results can be explained by the destabilizing effect of D266W substitution on the overall protein structure as was observed by CD, possibly caused by the increased size of the tryptophan residue, when compared to the native aspartic acid. D266Y bound to H3K4me2 and H3K4me3 with 9- and 10-fold decreased binding affinities relative to WT Sgf29. Enthalpy decreased markedly for both H3K4me2 (3.3 kcal mol^-1^) and H3K4me3 (3.3 kcal mol^-1^), but was only partially compensated by an increase in entropy of about 2.1 kcal mol^-1^ for both methylation states. D266F substitution displayed even greater reduction in binding affinities for H3K4me3 (14-fold) and H3K4me2 (12-fold). The observed decrease in Δ*H*° of 4.6 kcal mol^-1^ and 4.7 kcal mol^-1^ for H3K4me3 and H3K4me2, respectively, was only partially compensated by more favorable -TΔ*S*° (3.1 kcal mol^-1^) for both peptides. Observations that hydrophobic Tyr and Phe substituents at the D266 site exhibit more favorable entropy of binding relative to native D266 possibly derives from more favorable desolvation of the aromatic amino acids and a higher degree of flexibility of the protein or histone in the complex. Collectively, mutagenesis data on Sgf29 show that the presence of aromatic residues that have a potential to form a full cage-like module does not exhibit advantage for binding of the natural sequence of H3K4me2/3 over the half-cage arrangement. Appart from the structural changes observed by CD, the increase in bulk when exchangeing aspartic acid for the aromatic residues phenylalanine, tyrosine or tryptophan may also affect binding affinity due to steric hindrance, which may have been introduced into the aromatic cage.

Together with recent binding studies on BPTF, L3MBTL1, TAF3, and HP1 reader proteins our study suggests that the underlying mechanisms for the specific recognition of Kme2 by negatively-charged Asp or Glu vary in each case, and that the recognition does not obey an involvement of the same type of favorable interactions. For instance, BPTF Y17E variant exhibits selectivity for binding to Kme2 over Kme3 due to direct H-bonding with hydrogen of ^+^NHMe_2_ side-chain[16]. L3MBTL1, a reader domain protein that specifically recognizes lower methylation states of lysine (i.e. Kme and Kme2), also makes a direct H-bond with negatively-charged D355 located in the recognition site. In addition, D877 residue of TAF3 is not directly involved in intermolecular interactions with the Kme2 side-chain, but forms a hydrogen bond with the hydroxyl group of Thr6 in the H3K4me2 peptide[17]. In contrast, E52 residue of chromodomain HP1 interacts with the positively-charged ^+^NHMe_2_ via water-mediated H-bonding[18].

## Conclusions

Our thermodynamic results on Sgf29’s recognition site variants, supported by crystallographic analyses, suggest that favorable H-bonding interaction between D266 and ^+^NHMe_2_ is unlikely to be present, thus emphasizing the existence of favorable direct electrostatic interactions between negatively-charged D266 and positively-charged Kme2 or Kme3. The magnitude of these interactions for associations with Kme2 and Kme3 is similar, which results in comparable selectivities (1.3–1.9) for the Sgf29 variants that were investigated. Additionally, our work suggests that the negative charge on the D266 residue appears to play an important role in maintaining the structural integrity of the loop region that constitutes one part of the aromatic cage of Sgf29 in its apo state. We furthermore showed that H-bonding between Y245 and backbone K4 amide provides an additional driving force (~1 kcal mol^-1^) for the specific readout of H3K4me2/3 by Sgf29. This work, together with our previous studies that confirmed the existence of the H3A1 recognition site,[12] highlights the importance of electrostatic interactions and hydrogen bonding in specific recognition of H3K4me2/3 by the Sgf29 tandem tudor domain.

## Experimental Section

### Solid-phase peptide synthesis

Fmoc-protected dimethyllysine and trimethyllysine were synthesized by modifications of reported procedures[12]. The ARTKme3QTARKS and ARTKme2QTARKS peptides were made on solid phase by using manual solid phase peptide synthesis applying Fmoc chemistry. Peptides containing a carboxylic acid at the C terminus were made on Wang resin (± 0.6 mmol g^-1^) and couplings were done in DMF with Fmoc-protected amino acid (3.0 equiv.), diisopropylcarbodiimide (DIPCDI) (3.3 equiv.) and hydroxybenzotriazole (HOBt) (3.6 equiv.). Completion of the reaction was determined with the Kaiser test, and removal of Fmoc was achieved by treatment with a large excess of piperidine (20%) in DMF for 20–30 min. Every wash step was performed with 3 × DMF and after building completion the Fmoc was removed followed by wash 3 × DMF and 3 × Et_2_O continued by drying of the resin in vacuo. The peptide (including acid-labile protecting groups) were cleaved by a mixture of TFA (92.5%), H_2_O (2.5%), tri-isopropylsilane (TIPS) (2.5%) and ethane-1,2-dithiol (EDT) (2.5%). After mixing / shaking for 4–5 h, the product was precipitated in Et_2_O, and the Et_2_O was decanted off after centrifugation (3500 rpm, 3 min, Hermle 220.72 v04). Peptides were identified by LC-MS and MALDI-TOF. Purification was performed on preparative HPLC.

### Site directed mutagenesis

Primers were designed using PrimerX software (http://bioinformatics.org/primerx/) and obtained from Biolegio (S1 Table). After DNA amplification by PCR, the reaction mixtures were subjected to Dpn1 treatment and the reaction products were subsequently transformed into competent *E*.*coli* top 10 cells. After DNA isolation, the presence of the desired mutation was verified by sequence analysis.

### Protein expression and purification

Wild-type and variant Sgf29 reader domain proteins (Homo sapiens, residues 115–293) were expressed in terrific broth (TB) medium containing 50 μg ml^-1^ kanamycin, using *E*. *coli* Rosetta BL21 DE3 PlysS as expression host. Bacteria were cultured to OD_600_ ≈ 0.6 at 37°C, 200 rpm after which the cultures were induced using 1 mM IPTG (final conc.). Cultures were then incubated at 16°C for 16 hours. Cells were harvested and lysed by sonication in 1x PBS, 250 mM NaCl, 5% glycerol, 0.2% CHAPS, 10mM β-mercaptoethanol. Lysate was centrifuged and the supernatant purified using Ni-NTA beads. The 6xHis tag was cleaved off overnight at 4°C using TEV-protease. Size exclusion chromatography, using a superdex 75 column, was employed as a final purification step with 25 mM TRIS pH 8.0, 50 mM NaCl, 1 mM DTT as eluent. Protein concentrations were determined by UV/Vis spectroscopy at 280 nm.

### CD Spectroscopy

CD measurements were conducted using a J-815 circular dichroism spectropolarimeter (JASCO) at 0.1 mg ml^-1^ protein concentration in a 1 mm cuvette using 10 mM NaH_2_PO_4_ pH 7.5 as buffer. The wavelength range scanned was 180–260 nm using a bandwidth of 1 nm at a time constant of 0.5 second and scan rate of 50 nm sec^-1^. Obtained spectra are averages of 10 measurements and have been smoothed using a Savitzky-Golay filter with a convolution width of 11.

### Differential Scanning Fluorimetry (DSF)

Sgf29 protein melt curves were obtained as described by Reinhard et. al. [19] using a StepOne-Plus Real-Time PCR system (Applied Biosystems) and MicroAmp fast optical 96-well reaction plates (Applied Biosystems). SYPRO-Orange protein gel stain (Invitrogen) was used as a reporter dye, emitting fluorescence in the FAM channel. Total reaction volume was 25 μl; 20 μl buffer (25 mM TRIS pH 7.5, 50 mM NaCl, 1 mM DTT), 2.5 μl of 25 μM protein and 2.5 μl SYPRO-Orange dye (diluted 1:100 in ddH_2_O). Melt curve data were obtained in triplicate, in a temperature range of 20–95°C at a step-wise temperature increment of 1°C min^-1^. Obtained data were analyzed using DSF Analysis v3.0.2 software, designed by Niesen et. al. (available via ftp://ftp.sgc.ox.ac.uk/pub/biophysics/) [20].

### Isothermal Titration Calorimetry (ITC)

ITC experiments were conducted using an automated Microcal AutoITC200 (GE Healthcare). Generally 1.2 mM of peptide was titrated to 100 μM of protein. For variants that displayed low binding affinities, concentrations of 2.4 mM peptide were titrated to 200 μM of protein. The buffers used for ITC experiments were the same as the elution buffer used for SEC; 25 mM TRIS pH 8.0, 50 mM NaCl, 1 mM DTT. Heats of dilution were subtracted from the titration binding data curve fitting. Curve fitting was performed with Origin 6.0 (Microcal Inc., Northampton, MA) using a one-site model.

## Supporting Information

S1 Fig MALDI-TOF spectrum of HPLC-purified ARTKme2QTARKS peptide. Calculated [M] 1173.69, found [M+H]^+^ 1174.6.
(TIF)

S2 Fig MALDI-TOF spectrum of HPLC-purified ARTKme3QTARKS peptide. Calculated [M] 1187.71, found [M+H]^+^ 1187.6.
(TIF)

S3 Fig A 15% SDS-PAGE gel displaying Ni-NTA purified recombinant WT Sgf29 and its variants. Amino acid substitutions are displayed in single letter code. From left to right: Sgf29 wild-type, D266A, D266E, D266F, D266N, D266W, D266Y, Y238F and Y245F, respectively.
(TIF)

S1 Table Primers used for Sgf29 mutagenesis. Mutations are underscored and codons are indicated in grey.
(DOCX)
