# Supplementary figures and images for: The Role of Electrostatic Interactions in Binding of Histone H3K4me2/3 to the Sgf29 Tandem Tudor Domain
Source: PLoS One. 2015 Sep 30;10(9):e0139205. doi: 10.1371/journal.pone.0139205 (PMC4589357; doi:10.1371/journal.pone.0139205)

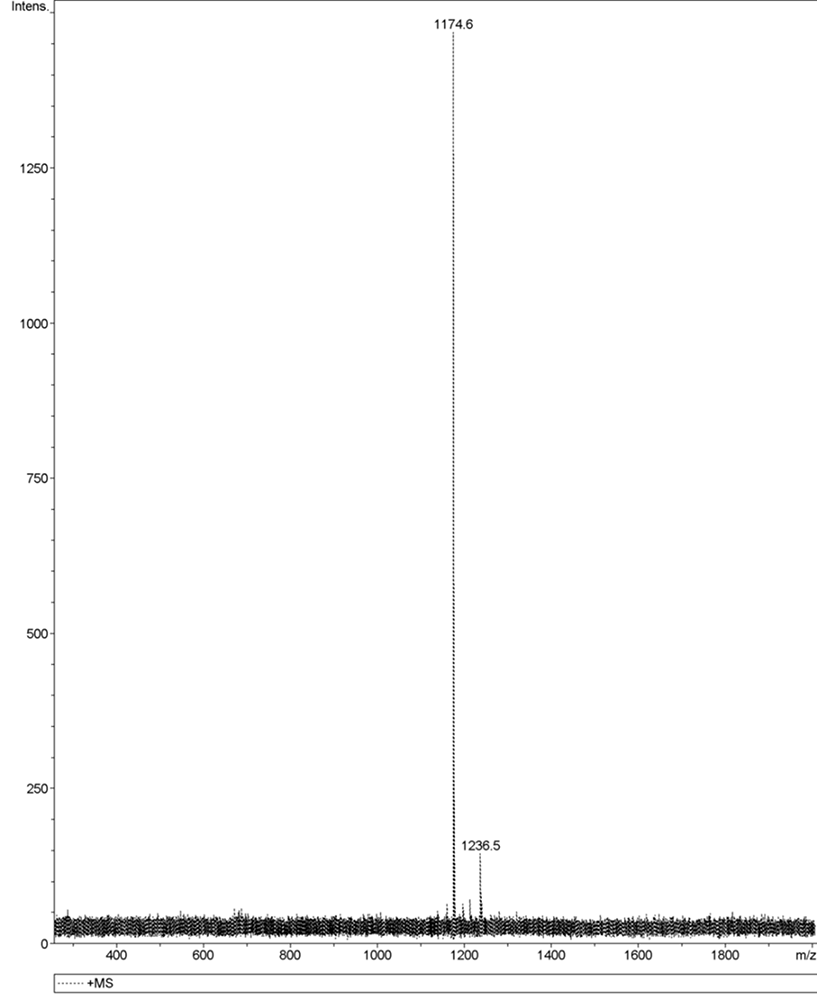

Supplement: S1 Fig — Calculated [M] 1173.69, found [M+H]+ 1174.6. (TIF) [file pone.0139205.s001.tif]

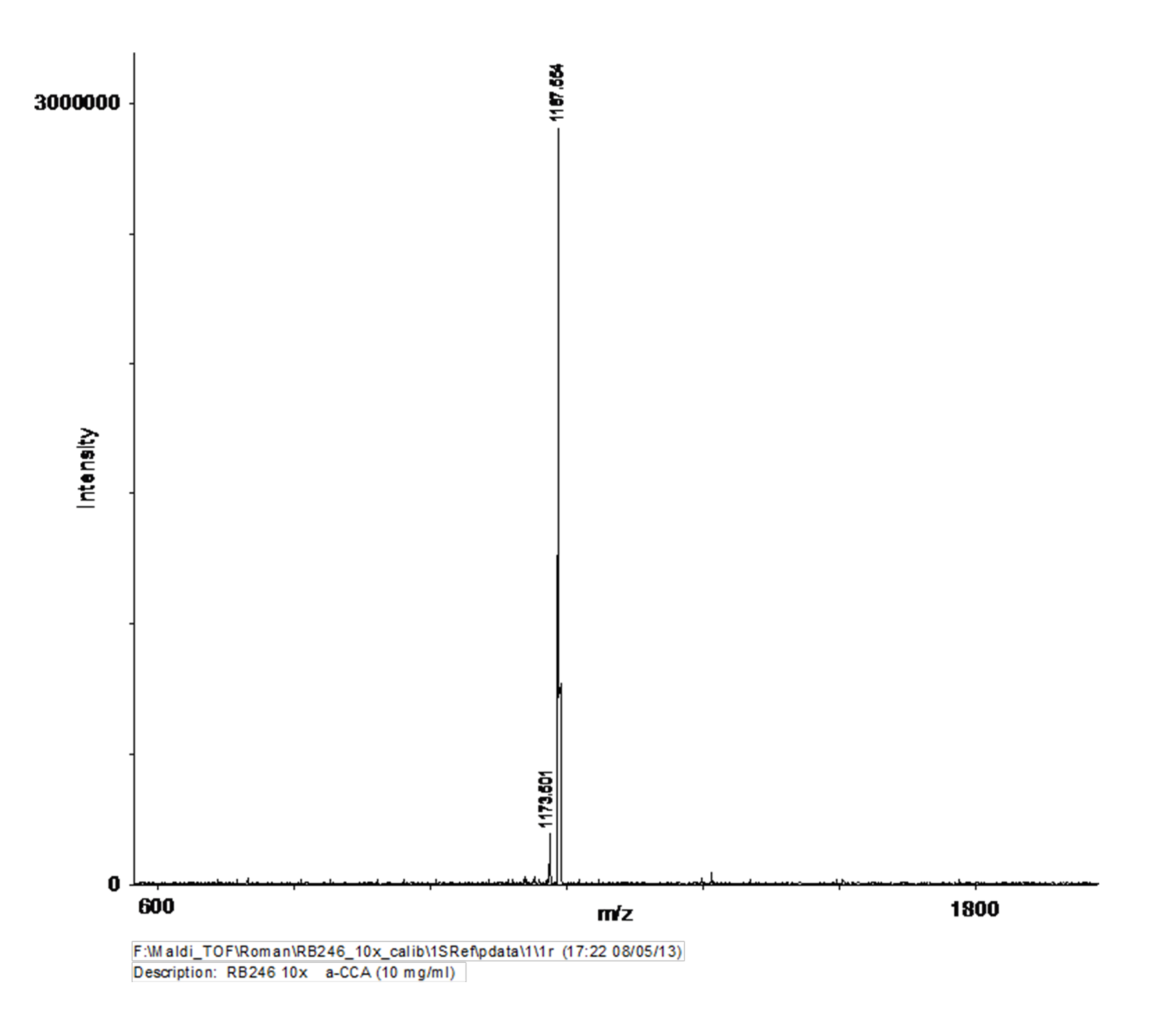

Supplement: S2 Fig — Calculated [M] 1187.71, found [M+H]+ 1187.6. (TIF) [file pone.0139205.s002.tif]

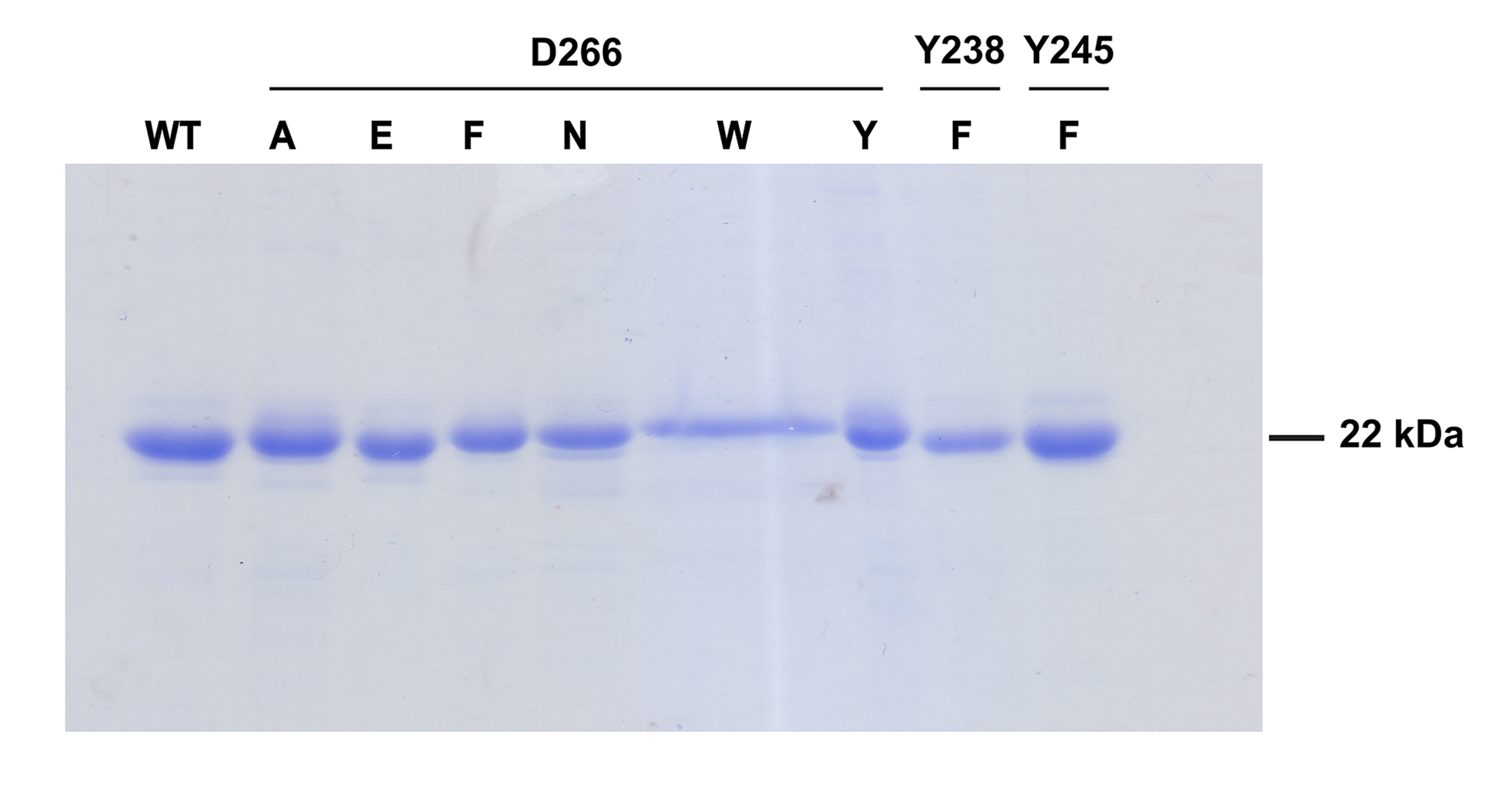

Supplement: S3 Fig — Amino acid substitutions are displayed in single letter code. From left to right: Sgf29 wild-type, D266A, D266E, D266F, D266N, D266W, D266Y, Y238F and Y245F, respectively. (TIF) [file pone.0139205.s003.tif]
