# Supplementary material for: The Role of Electrostatic Interactions in Binding of Histone H3K4me2/3 to the Sgf29 Tandem Tudor Domain
Source: PLoS One. 2015 Sep 30;10(9):e0139205. doi: 10.1371/journal.pone.0139205 (PMC4589357; doi:10.1371/journal.pone.0139205)
Supplement: S1 Table — Mutations are underscored and codons are indicated in grey. (DOCX) [file pone.0139205.s004.docx]

**S1 Table. Primers used for Sgf29 mutagenesis.** Mutations are underscored and codons are indicated in grey.

| **Sgf29 mutation** | **Primer sequence** |
| --- | --- |
| **D266E fwd.** | 5' CGGTCCTGTTTGAAGAAACCTCCTATGCAGATGG 3' |
| **D266E rev.** | 5' CCATCTGCATAGGAGGTTTCTTCAAACAGGACCG 3' |
| **D266A fwd.** | 5' CGGTCCTGTTTGAAGCCACCTCCTATGCAG 3' |
| **D266A rev.** | 5' CTGCATAGGAGGTGGCTTCAAACAGGACCG 3' |
| **D266N fwd.** | 5' CTCGGTCCTGTTTGAAAACACCTCCTATGCAG 3' |
| **D266N rev.** | 5' CTGCATAGGAGGTGTTTTCAAACAGGACCGAG 3' |
| **D266W fwd.** | 5' TACTCGGTCCTGTTTGAATGGACCTCCTATGCAGATGGC 3' |
| **D266W rev.** | 5' GCCATCTGCATAGGAGGTCCATTCAAACAGGACCGAGTAG 3' |
| **D266Y fwd.** | 5' CTCGGTCCTGTTTGAATACACCTCCTATGCAG 3' |
| **D266Y rev.** | 5' CTGCATAGGAGGTGTATTCAAACAGGACCGAG 3' |
| **D266F fwd.** | 5' CTACTCGGTCCTGTTTGAATTCACCTCCTATGCAGATGG 3' |
| **D266F rev.** | 5' CCATCTGCATAGGAGGTGAATTCAAACAGGACCGAGTAG 3' |
| **Y238F fwd.** | 5' CTGGCCCTGTTCCCCCAGACTAC 3' |
| **Y238F rev.** | 3' CACGACCGGGACAAGGGGGT 5' |
| **Y245F fwd.** | 5' ACCTGCTTCTTCCGCGCCCTG 3' |
| **Y245F rev.** | 3' GATGGACGAAGAAGGCGCGG 5' |
